# Effects of Physiologically Relevant Species of Organic Mercury on Mesenchymal Stem Cells and Neural Precursor Cells

**DOI:** 10.3390/metabo15120794

**Published:** 2025-12-14

**Authors:** Juliane Hostert, Nathalia Kirsten, Larissa Lührs, Ana Carolina Irioda, Izonete Cristina Guiloski, Katherine Athayde Teixeira de Carvalho, Cláudia Sirlene Oliveira

**Affiliations:** 1 Instituto de Pesquisa Pelé Pequeno Príncipe, Curitiba 80250-060, PR, Brazil; 2 Faculdades Pequeno Príncipe, Curitiba 80230-020, PR, Brazil

**Keywords:** methylmercury, stem cells, neurotoxicity

## Abstract

Background/Objectives: Methylmercury (MeHg) is a well-known environmental neurotoxic agent with significant detrimental effects on human health, particularly targeting the central nervous system. This study aimed to evaluate the impact of physiologically relevant species of MeHg, specifically MeHg–cysteine and MeHg–glutathione, on mesenchymal stem cells (MSCs) and neural precursor cells (NPCs). Methods: The NPCs were differentiated from the MSCs after being seeded on a natural functional biopolymer matrix. The cells were exposed to 0, 0.01, 0.5, 1.5, and 2.0 µM MeHgCl or its physiologically relevant species. Biochemical markers, including superoxide dismutase (SOD), glutathione peroxidase (GPx), glutathione S-transferase (GST), and reduced glutathione (GSH), were analyzed. Results: MeHgCl and its physiological species did not affect MSC viability. However, 1.5 and 2.0 µM MeHgCl caused a significant reduction (~25%) in NPC viability. SOD activity and GSH levels were not significantly altered in either MSCs or NPCs. In contrast, MeHg–glutathione (2.0 µM) significantly decreased GPx activity in both MSCs (~62%) and NPCs (~78%). GST activity remained unchanged in MSCs, but NPCs showed a significant decrease (~50%) after exposure to 1.5 and 2.0 µM MeHg–glutathione. Conclusions: The results indicate that MSCs are more resistant to MeHg toxicity, whereas NPCs display markedly susceptibility. These findings highlight the distinct cellular responses to MeHg exposure. The disruption of antioxidant defenses, particularly in NPCs, may promote oxidative stress and ultimately lead to cell death.

## 1. Introduction

Mercury (Hg) is a toxic environmental pollutant, and its organic form, methylmercury (MeHg), is a well-known neurotoxic agent. MeHg can cause extensive damage to human health, predominantly manifesting as memory and learning disabilities and progressive paralysis [1,2,3].

Fetuses and developing children are the most vulnerable populations in terms of exposure to MeHg [4]. They are susceptible to nervous system alterations due to exposure to MeHg, even if the mother has no obvious symptoms [4,5]. The blood–brain barrier is a highly selective structure that protects the brain against potentially harmful substances in the bloodstream. However, MeHg has characteristics that allow it to pass through this barrier, such as forming complexes with cysteine [3,5,6]. The MeHg–cysteine complex mimics the structure of an essential amino acid, methionine, which is normally transported to the brain through amino acid transporters in the blood–brain barrier [2,6]. The same pattern is observed in the placental barrier [7,8].

The central nervous system (CNS) is one of the most complex and essential structures in the human body, responsible for processing information and coordinating a wide range of vital functions, including movement, sensory perception, behavior regulation, and cognitive processing [9]. Understanding the development and functioning of the CNS is fundamental to neuroscience, particularly in the investigation of neurological and neurodegenerative diseases [10,11].

To study neurodevelopment, cellular models such as mesenchymal stem cells (MSCs) and neural precursor cells (NPCs) are widely used. MSCs, derived from tissues such as bone marrow and adipose tissue, can differentiate into multiple cell types, including osteocytes, adipocytes, and chondrocytes [12,13]. Furthermore, these cells have shown the potential to differentiate into neurons under certain experimental conditions. NPCs, on the other hand, are cells with an intrinsic capacity to specifically differentiate into neural cells, making them a valuable tool for studying the processes of neurogenesis and neural development [14,15].

The studies of Patnaik et al. [16], Huang et al. [17], de Souza-Rodrigues et al. [18], and Torres-Ruiz et al. [19] demonstrated the toxic effects of MeHg (0.1 to 5 µM) to stem cells and their derivatives. These studies consistently showed that MeHg alters cell viability and increases oxidative stress. However, few studies explored the effects of the physiologically relevant species of MeHg, namely MeHg–cysteine and MeHg–glutathione in cell culture, more specifically CHO-k1 (rat ovary) [20], C6 (rat glioma) [21], and BeWo (human placenta) [8]. In this context, the present study aims to investigate the impacts of physiologically relevant species of MeHg on cell viability and biochemical markers of human MSCs and their NPC derivatives.

## 2. Materials and Methods

### 2.1. Experimental Design

**Figure 1:**
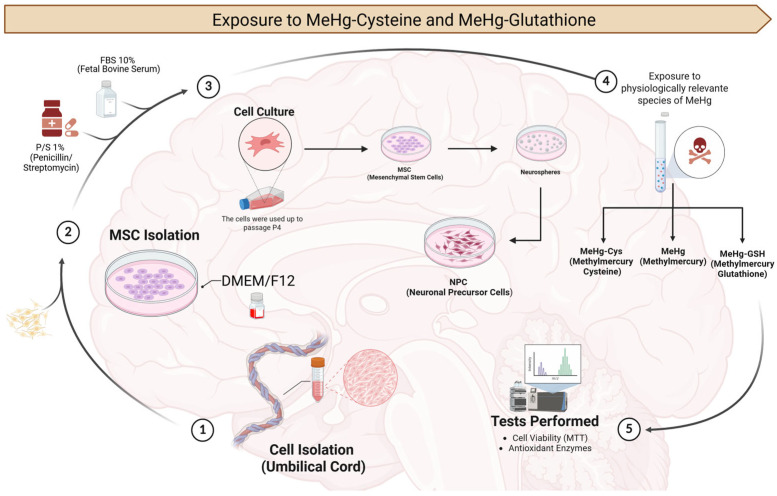
Experimental design. The process of isolation and cultivation of MSCs and NPCs was followed by the exposure to physiologically relevant species of MeHg [MeHg–cysteine (MeHg-Cys) and MeHg–glutathione (MeHg-GSH)]. After exposure, the cell viability and biochemical markers were evaluated.

MSCs and their derivatives NPCs, isolated from the Wharton’s Jelly of umbilical cords, were exposed to physiologically relevant species of MeHg (MeHg–cysteine and MeHg–glutathione) for 24 h. After the exposure, the cellular viability and biochemical markers were evaluated (Figure 1).

#### 2.1.1. MSCs and NPCs

MSCs and NPCs were produced and supplied by the Laboratory of Advanced Therapies and Cellular Biotechnology at the Pelé Pequeno Príncipe Research Institute, under the direction of Prof. Dr. Katherine Athayde Teixeira de Carvalho. All samples were collected and handled following good manufacture practices. This project was approved by the Human Ethics and Research Committee of Faculdades Pequeno Príncipe, numbered: 3.288.245 (29 April 2019). Briefly, MSCs were isolated from Wharton’s Jelly of umbilical cords as described by Stricker et al. [22] and Dobuchak et al. [23]. After collection and isolation, the cells were seeded on polypropylene flasks with a flat area of 75 cm^2^ with cultured in Dulbecco’s Modified Eagle’s Medium/nutrient Mixture F12 (DMEM-F12; Sigma-Aldrich^®^-USA, St. Louis, MO, USA) supplemented with 10% fetal bovine serum (FBS-Gibco^®^, Thermo Fisher^®^, Waltham, MA, USA), and 1% antibiotic (100 IU/mL penicillin, 0.1 mg/mL streptomycin-Thermo Fisher^®^, Waltham, MA, USA), and incubated at 37 °C and 5% CO_2_. The culture medium was changed twice a week until the cells reached 80 to 90% confluency. After expansion, the MSCs were subjected to adipogenic, osteogenic, and chondrogenic differentiation (trilineage differentiation) as described by Oliveira et al. [24], Rosa et al. [25], and Perussolo et al. [26].

NPCs were obtained from the MSCs after neurosphere formation. The formation of the neurospheres was carried out using a protocol developed by the Laboratory of Advanced Therapies and Cellular Biotechnology of Prof. Dr. Katherine Athayde Teixeira de Carvalho, without the use of growth or induced factors, as described in Dobuchak et al. [23]. After the formation of the neurospheres, they were collected and cultivated for NPCs expansion as described by Stricker et al. [22].

Both MSCs and NPCs were characterized by flow cytometry regarding the presence or absence of cell markers as determined by the International Society for Cellular Therapy. The analyses were carried out as described by Dobuchak et al. [23] and Irioda et al. [27].

#### 2.1.2. Exposure to the Physiologically Relevant Species of MeHg (MeHg–cysteine and MeHg–glutathione)

The physiologically relevant species of MeHg (MeHg–cysteine and MeHg–glutathione) were prepared in a 1:2 solution MeHg (Sigma-Aldrich^®^-USA)/cysteine (VETEC–Br, Vila Olímpia, SP, Brazil) or reduced glutathione (GSH; Sigma-Aldrich^®^-USA) as described by Bridges and Zalups [28]. MSCs and NPCs were seeded into 6- and 48-well plates at densities of 1 × 10^4^, 5 × 10^4^, 2 × 10^5^, or 1 × 10^6^ cells/well, depending on the assay performed. Cells were exposed to 0.01–2.0 μM MeHgCl, MeHg–cysteine, or MeHg–glutathione for 24 h. The concentration–response relationships and exposure durations were established in preliminary experiments conducted in our laboratory (data not shown).

### 2.2. Cell Viability Assay

After MeHg and its physiologically relevant species exposure, all treatment medium was removed, and 3-(4,5-dimethylthiazol-2-yl)-2,5-diphenyltetrazolium bromide (MTT; Invitrogen^®^-USA, Waltham, MA, USA) solution (5 mg/mL) was added and incubated at 37 °C for 3 h. After incubation, all supernatant was removed and 250 µL of dimethyl sulfoxide (DMSO) (Êxodo Cientifica^®^-Br, Sumaré, SP, Brazil) was added to dissolve the formazan crystals. The absorbance was read spectrophotometrically at 595 nm [29]. The results are expressed as percentages of the control.

### 2.3. Biochemical Analyses

#### 2.3.1. MSCs and NPCs Preparation

To perform the biochemical analyses, MSCs and NPCs exposed to MeHg and its physiologically relevant species, as described in Section 2.1.2, were previously prepared. Briefly, the culture media were collected, wells were washed with phosphate-buffered saline (PBS; Sigma-Aldrich^®^-USA), and the cells were harvested by trypsin (Sigma-Aldrich^®^-USA). To inactivate the action of trypsin, DMEM-F12 supplemented with 10% FBS and 1% penicillin/streptomycin was added. Then, the samples were centrifuged at 400× *g* for 5 min, and the supernatant was discarded. For the analysis of superoxide dismutase (SOD), glutathione peroxidase (GPx), glutathione S-transferase (GST), GSH, acetylcholinesterase (AChE), and total protein, the samples were resuspended in 600 μL of PBS. For the N-acetyl-β-D-glucosaminidase (NAG) analysis, the samples were resuspended in 300 μL of Hexadecyl Trimethyl Ammonium Bromide (HTAB) buffer (13,7 mM). The aliquots were stored at −80 °C until the biochemical analyses.

#### 2.3.2. SOD Activity

SOD activity was carried out according to the method proposed by Gao et al. [30] based on the ability of SOD to inhibit the auto-oxidation of pyrogallol. First, 40 μL of the sample resuspended in PBS, 1 M Tris (Sigma-Aldrich^®^-USA), 5 mM ethylenediaminetetraacetic acid (EDTA; VWR Life Science^®^-USA, Radnor, PA, USA) buffer, pH 8.0, and 15 mM pyrogallic acid (Labsynth^®^-Br, Diadema, SP, Brazil) were added to 2 mL microtubes. Then, the samples were incubated for 30 min in the dark at room temperature. After incubation, 1 N hydrochloric acid (Sigma-Aldrich^®^-USA) was added to stop the reaction, then 200 μL of each sample was transferred to 96-well microplates, and absorbance was measured spectrophotometrically at 440 nm. The results were expressed as U of SOD/μg of protein.

#### 2.3.3. GPx Activity

GPx activity was performed using the method of Paglia and Valentine [31]. In a 96-well microplate, 10 μL of the sample resuspended in PBS was pipetted in triplicate. Afterwards, 130 μL of the reaction solution 1 [0.1 M PBS, pH 7.0, 3.1 mM sodium azide (Labsynth^®^-Br), 0.31 mM NADPH (Sigma-Aldrich^®^-USA), 3.1 mM GSH, and 1.54 U/mL glutathione reductase (Sigma-Aldrich^®^-USA)] was added. After 2 min of incubation at room temperature, 60 μL of solution 2 (5 mM hydrogen peroxide, Sigma-Aldrich^®^-USA, and 0.1 M sodium phosphate buffer, pH 7.0) were added into the wells. The plate was incubated for 5 min at room temperature. The absorbance was measured spectrophotometrically at 340 nm once a minute for 5 min. The GPx activity was expressed in nmol/min/μg of protein.

#### 2.3.4. GST Activity

GST activity was evaluated using the method described by Keen et al. [32]. First, 20 μL of the sample, resuspended in PBS, and 180 µL reaction solution [3.0 mM 1-chloro-2,4-dinitrobenzene (CDNB; Sigma-Aldrich^®^-USA), 3.0 mM GSH, and 0.1 M phosphate buffer, pH 6.5] were added into a 96-well microplate. The plate was incubated for 5 min, and absorbance was measured spectrophotometrically at 340 nm. GST activity was expressed as nmol/min/μg of protein.

#### 2.3.5. AChE Activity

AChE activity was measured by the method described by Ellman et al. [33]. In a 96-well plate, 25 µL of the sample resuspended in PBS, 200 µL of 0.75 mM 5,5-dithio-bis-(2-nitrobenzoic acid) (DTNB; Sigma-Aldrich^®^-USA), and 50 µL of 10 mM acetylthiocholine (ACh; Sigma-Aldrich^®^-USA) were added. The absorbance was measured spectrophotometrically at 405 nm once a minute for 5 min. The activity was expressed as nmol ACh/min/μg of protein.

#### 2.3.6. GSH Levels

GSH concentration was measured using the method proposed by Sedlak and Lindsay [34]. First, 100 μL of samples were reserved in microtubes to which 25 μL of 50% trichloroacetic acid (Labsynth^®^-Br) was added. These were vortexed and centrifuged at 12,000× *g* for 10 min at 4 °C, then 50 μL of each sample was added in duplicate to 96-well microplates, along with 230 μL of Tris-base buffer (400 mM; pH 8.9) and 20 μL of DTNB (Sigma-Aldrich^®^-USA) at 2.5 mM (in 25% methanol (Êxodo Científica^®^-Br, Sumaré, SP, Br) in 400 mM Tris-base buffer, pH 8.9) were added. GSH concentration was determined by comparison with a GSH standard curve (0–80 μg/mL). Readings were taken at 415 nm, and results were expressed as μg GSH/μg protein.

#### 2.3.7. NAG Activity

NAG activity was performed based on the method proposed by Bailey [35]. In a 96-well microplate, 25 μL of the sample resuspended in HTAB (Sigma-Aldrich^®^-USA), 25 μL of 4-Nitrophenyl N-acetyl-β-D-glucosaminide solution (Sigma-Aldrich^®^-USA; 3.44 mM), and 100 μL of citrate buffer (Sigma-Aldrich^®^-USA; 50 mM and pH 4.5) were added. The microplate was incubated for 60 min, and then 100 μL of glycine buffer (Sigma-Aldrich^®^-USA; 200 mM and pH 10.4) was added. The reading was carried out by spectrophotometry at a wavelength of 405 nm. The activity was expressed as optical density (OD)/μg of protein.

#### 2.3.8. Total Protein Levels

Total protein content was determined according to the Bradford assay [36] and used to normalize the enzymatic activities detailed in the preceding sections. Briefly, 10 µL of the sample resuspended in PBS and 250 µL of Bradford reagent (Sigma-Aldrich^®^-USA) were pipetted into the 96-well plates. A standard curve of bovine serum albumin (BSA; Sigma-Aldrich^®^-USA) was prepared (0–1 mg BSA/mL). The absorbance was measured spectrophotometrically at 595 nm. Total protein was calculated as μg of protein/mL.

### 2.4. Statistical Analysis

For all experiments, at least three independent biological replicates were performed using three independent MSC isolates (and NPCs derivatives). The data were statistically analyzed using the GraphPad Prism software, version 5.0, using the Kruskal–Wallis test followed by Dunn’s test. The choice of the non-parametric test was defined after carrying out the Shapiro normality distribution test. To better visualize the data, the results were presented as the mean ± standard error of the mean (SEM). Results were considered statistically significant when *p* < 0.05.

## 3. Results

### 3.1. MSCs and NPCs Characterization

The results of trilineage differentiation are presented in Figures S1–S3 of Supplementary Materials. The MSCs differentiated into adipogenic, osteogenic, and chondrogenic cells. After the trilineage differentiation, the NPCs were obtained through MSCs neurospheres formation (Figure S4 of Supplementary Materials). Microscopically, no morphological differences were observed between MSCs and NPCs (Figure S5 of the Supplementary Materials). However, Stricker et al. [22] demonstrated that NPCs express neural cell markers, such as *GFAP*, *MAP2*, and *βIII-tubulin*, whereas MSCs do not. Regarding flow cytometry characterization, MSCs and NPCs showed positivity for CD13, CD90, CD105, and CD73 above 92%. Regarding CD34, CD45, and HLA-DR, the samples showed negative staining and weak positivity for HLA-ABC (Figure S6 of Supplementary Materials).

### 3.2. Cell Viability

**Figure 2:**
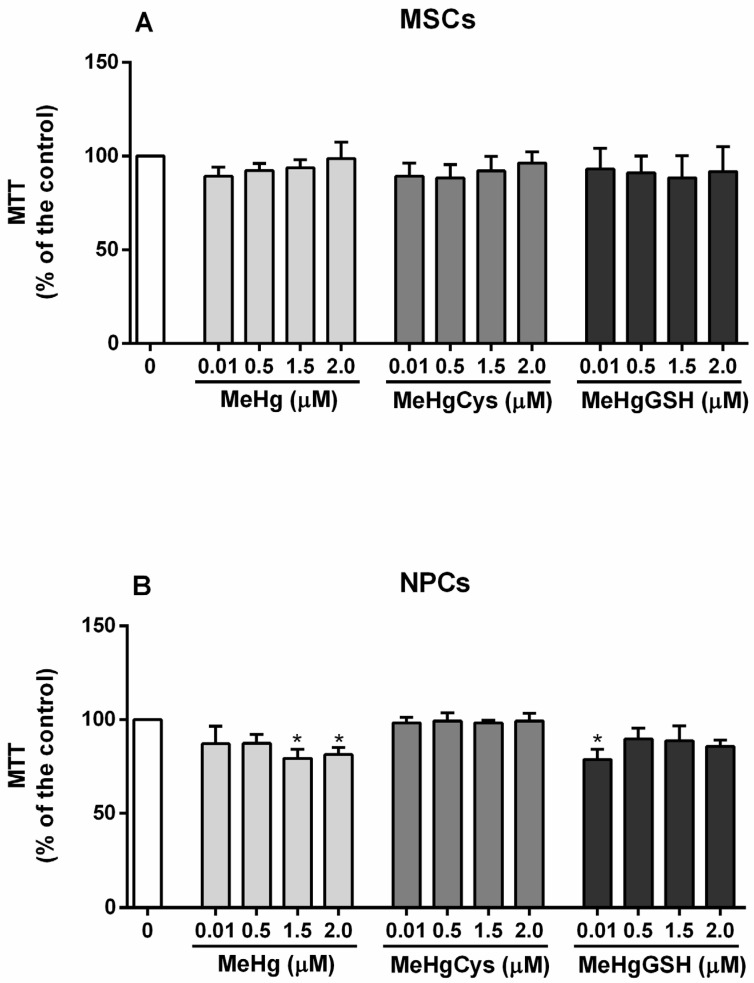
Mesenchymal stem cells (MSCs; (**A**)) and neural precursor cells (NPCs; (**B**)) viability. Cells were exposed for 24 h to MeHg, MeHg–cysteine (MeHgCys), or MeHg–glutathione (MeHgGSH). The results were analyzed by the Kruskal–Wallis test followed by Dunn’s post-test and presented as mean ± SEM (n = 3). * means statistically significant differences from the control group (0; not exposed cells).

The viability of the MSCs and NPCs exposed to the physiologically relevant species of MeHg is depicted in Figure 2. The Kruskal–Wallis test revealed the absence of MeHg, MeHg–cysteine, and MeHg–glutathione effects on MSCs viability. In contrast to MSCs, the Kruskal–Wallis test revealed the effect of MeHg and its physiologically relevant species on NPCs viability [H(13) = 21.57; *p* = 0.0426]. In fact, the highest concentration tested of MeHg (1.5 and 2.0 μM) resulted in a significant decrease in NPCs viability (~25%). Interestingly, only the lowest concentration tested of MeHg–glutathione (0.5 μM) decreased the NPCs viability (~25%). In the case of MeHg–cysteine, no alterations were observed in NPCs viability.

### 3.3. Biochemical Analyses

#### 3.3.1. SOD Activity

**Figure 3:**
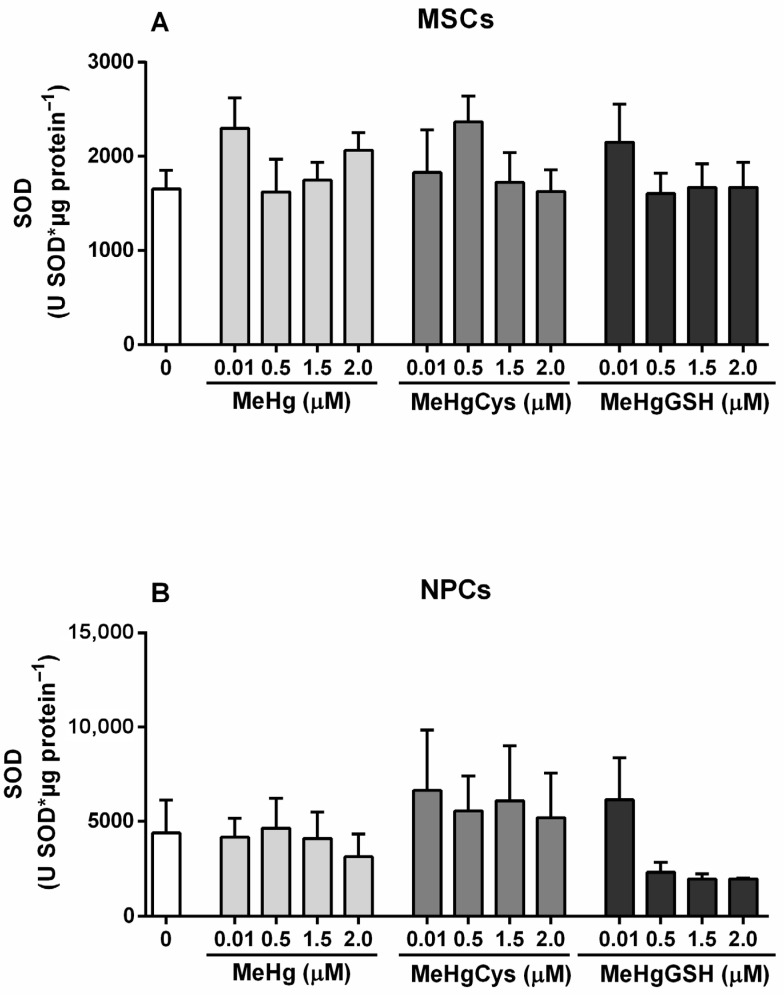
Mesenchymal stem cells (MSCs; (**A**)) and neural precursor cells (NPCs; (**B**)) SOD activity. Cells were exposed for 24 h to MeHg, MeHg–cysteine (MeHgCys), or MeHg–glutathione (MeHgGSH). The results were analyzed by the Kruskal–Wallis test followed by Dunn’s post-test and presented as mean ± SEM (n = 6–9).

The SOD activity of the MSCs and NPCs exposed to the physiologically relevant species of MeHg is depicted in Figure 3. The Kruskal–Wallis test revealed the absence of effects of MeHg and its physiologically relevant species on the SOD activity in both MSCs and NPCs. Although not statistically significant, NPCs displayed a marked reduction (~55%) in SOD activity with increasing concentrations of MeHg–glutathione.

#### 3.3.2. GPx Activity

**Figure 4:**
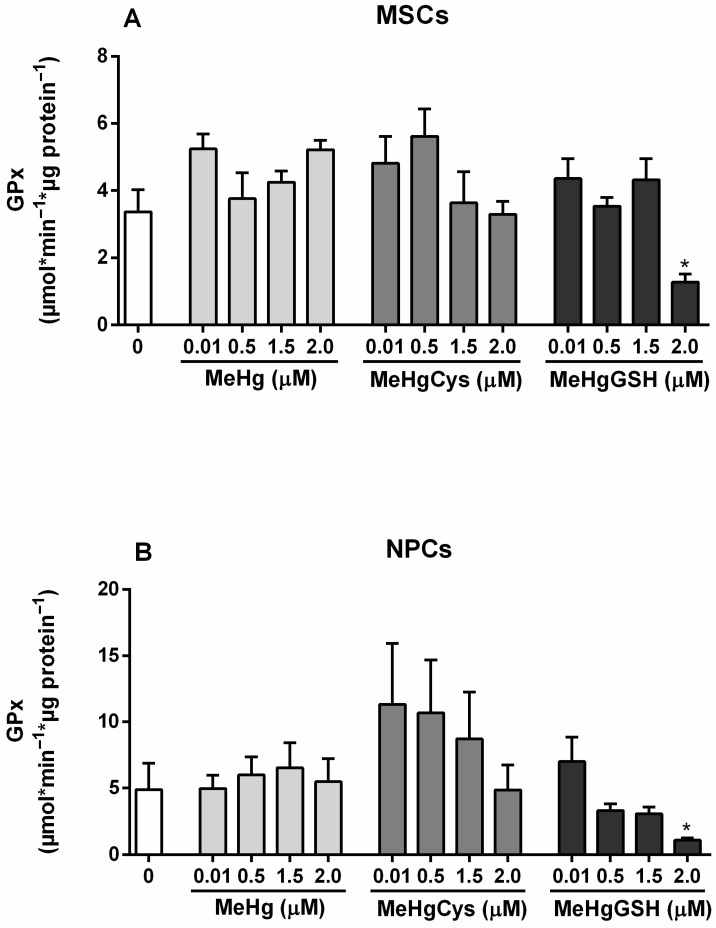
Mesenchymal stem cells (MSCs; (**A**)) and neural precursor cells (NPCs; (**B**)) GPx activity. Cells were exposed for 24 h to MeHg, MeHg–cysteine (MeHgCys), or MeHg–glutathione (MeHgGSH). The results were analyzed by the Kruskal–Wallis test followed by Dunn’s post-test and presented as mean ± SEM (n = 6–9). * means statistically significant differences from the control group (0; not exposed cells).

The GPx activity of the MSCs and NPCs exposed to the physiologically relevant species of MeHg is depicted in Figure 4. The Kruskal–Wallis test revealed the effect of MeHg and its physiologically relevant species on MSCs GPx activity [H(13) = 31.10; *p* = 0.0019]. The highest concentration tested of MeHg–glutathione (2.0 μM) resulted in a significant decrease in MSCs GPx activity (~62%). Regarding NPCs, the Kruskal–Wallis test revealed the effect of MeHg and its physiologically relevant species on enzyme activity [H(13) = 28.64; *p* = 0.0045]. The highest concentration tested of MeHg–glutathione (2.0 μM) resulted in a significant decrease in NPCs GPx activity (~78%). Although not statistically significant, NPCs exposed to 0.01–1.5 μM MeHg–cysteine showed a tendency to increase GPx activity (>50%).

#### 3.3.3. GST Activity

**Figure 5:**
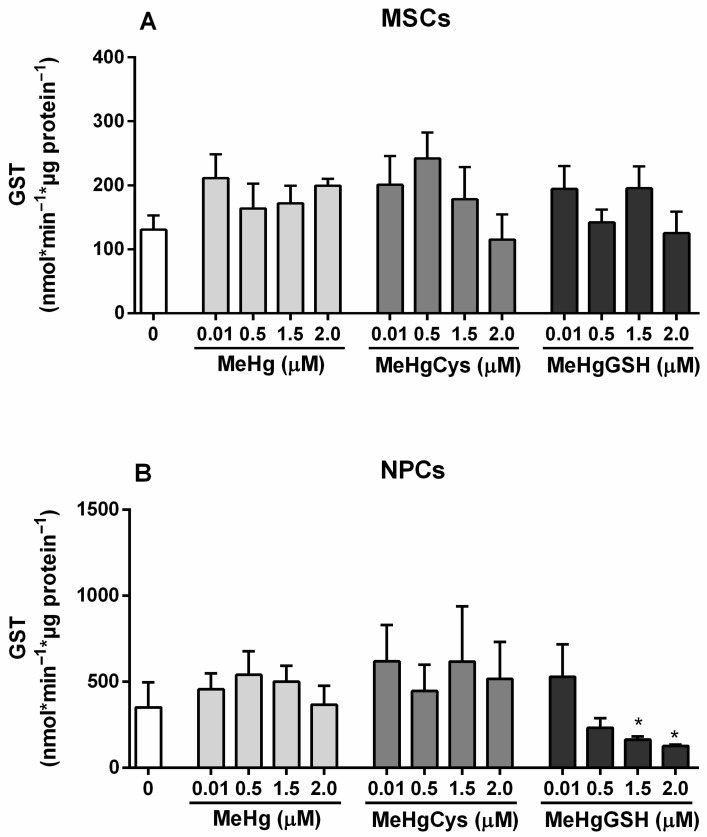
Mesenchymal stem cells (MSCs; (**A**)) and neural precursor cells (NPCs; (**B**)) GST activity. Cells were exposed for 24 h to MeHg, MeHg–cysteine (MeHgCys), or MeHg–glutathione (MeHgGSH). The results were analyzed by the Kruskal–Wallis test followed by Dunn’s post-test and presented as mean ± SEM (n = 6–9). * means statistically significant differences from the control group (0; not exposed cells).

The GST activity of the MSCs and NPCs exposed to the physiologically relevant species of MeHg is depicted in Figure 5. The Kruskal–Wallis test revealed the absence of MeHg and its physiologically relevant species on MSCs GST activity. Regarding NPCs, the Kruskal–Wallis test revealed the effect of MeHg and its physiologically relevant species on GST activity [H(13) = 21.49; *p* = 0.0437]. The highest concentration tested of MeHg–glutathione (1.5 and 2.0 μM) resulted in a significant decrease in NPCs GST activity (~50%).

#### 3.3.4. AChE Activity

**Figure 6:**
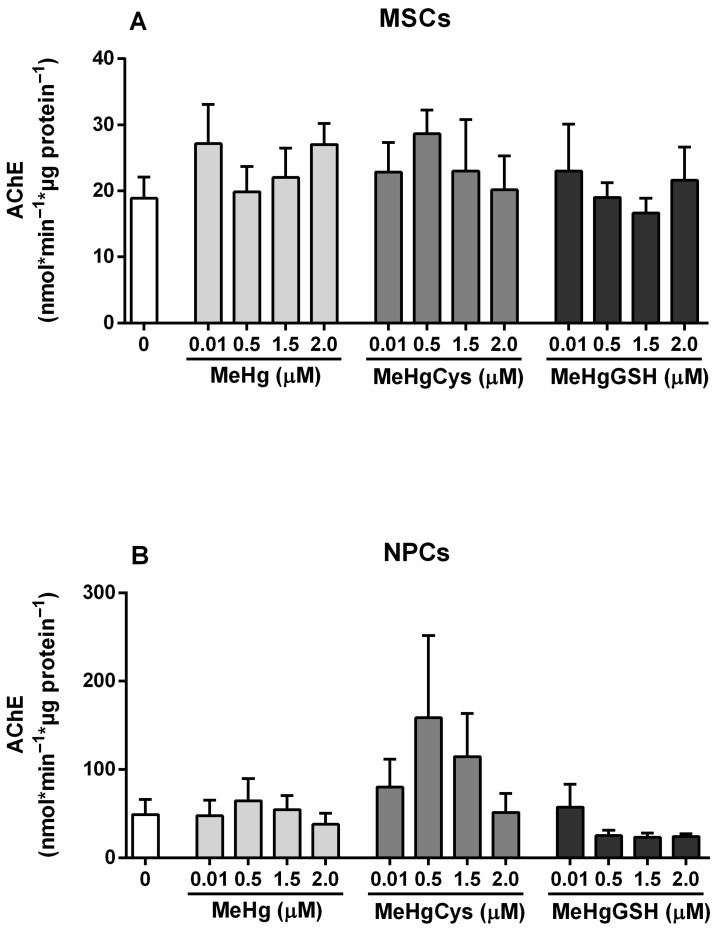
Mesenchymal stem cells (MSCs; (**A**)) and neural precursor cells (NPCs; (**B**)) AChE activity. Cells were exposed for 24 h to MeHg, MeHg–cysteine (MeHgCys), or MeHg–glutathione (MeHgGSH). The results were analyzed by the Kruskal–Wallis test followed by Dunn’s post-test and presented as mean ± SEM (n = 6–9).

The AChE activity of the MSCs and NPCs exposed to the physiologically relevant species of MeHg is depicted in Figure 6. The Kruskal–Wallis test revealed the absence of MeHg and its physiologically relevant species effects on the AChE activity in MSCs and NPCs. Although not statistically significant, NPCs exposed to MeHg–cysteine showed an increase in the AChE activity (>66%) and the exposure to MeHg–glutathione showed a tendency to inhibit the enzyme activity (~50%).

#### 3.3.5. GSH Levels

**Figure 7:**
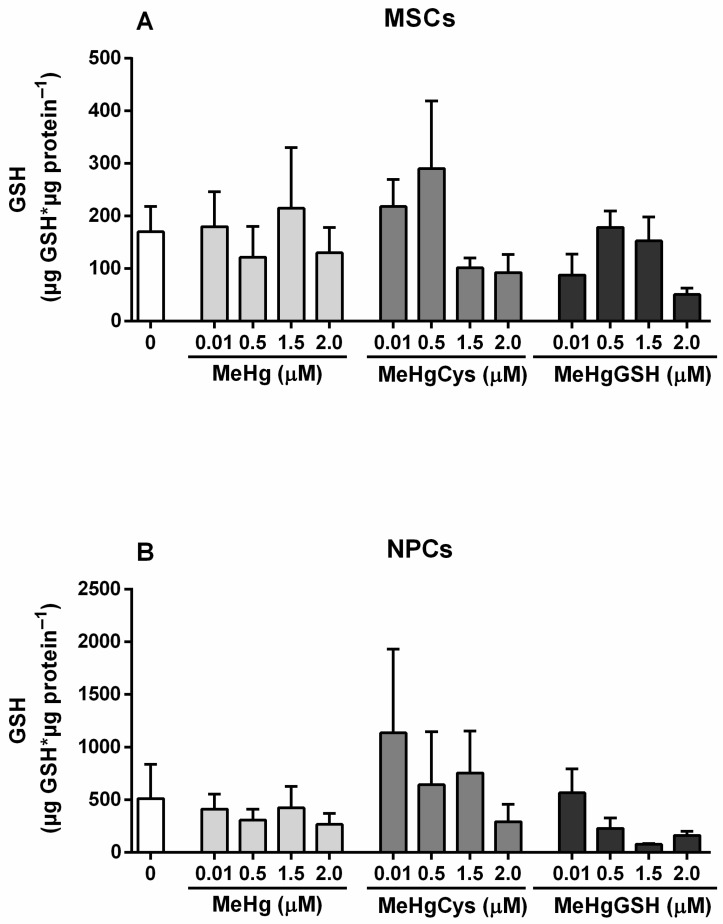
Mesenchymal stem cells (MSCs; (**A**)) and neural precursor cells (NPCs; (**B**)) GSH levels. Cells were exposed for 24 h to MeHg, MeHg–cysteine (MeHgCys), or MeHg–glutathione (MeHgGSH). The results were analyzed by the Kruskal–Wallis test followed by Dunn’s post-test and presented as mean ± SEM (n = 6–9).

GSH levels of the MSCs and NPCs exposed to the physiologically relevant species of MeHg are depicted in Figure 7. The Kruskal–Wallis test revealed the absence of MeHg and its physiologically relevant species effects on the GSH levels in both MSCs and NPCs.

#### 3.3.6. NAG Activity

**Figure 8:**
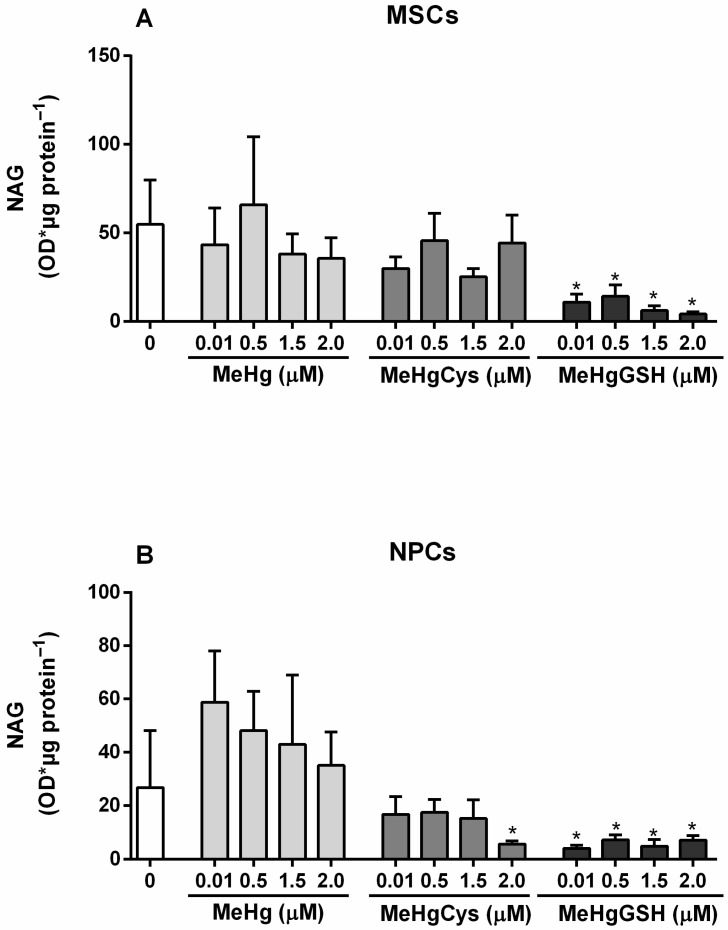
Mesenchymal stem cells (MSCs; (**A**)) and neural precursor cells (NPCs; (**B**)) NAG activity. Cells were exposed for 24 h to MeHg, MeHg–cysteine (MeHgCys), or MeHg–glutathione (MeHgGSH). The results were analyzed by the Kruskal–Wallis test followed by Dunn’s post-test and presented as mean ± SEM (n = 6–9). * means statistically significant differences from the control group (0; not exposed cells).

The NAG activity of the MSCs and NPCs exposed to the physiologically relevant species of MeHg is depicted in Figure 8. The Kruskal–Wallis test revealed the effect of MeHg and its physiologically relevant species on MSCs on NAG activity [H(13) = 29.94; *p* = 0.0029]. All the concentrations tested of MeHg–glutathione (0.01–2.0 μM) resulted in a significant decrease in MSCs NAG activity (~75%). Regarding NPCs, the Kruskal–Wallis test revealed the effect of MeHg and its physiologically relevant species on NAG activity [H(13) = 36.70; *p* = 0.0002]. The exposure to 2.0 μM MeHg–cysteine and 0.01–2.0 μM MeHg–glutathione significantly inhibited (>73%) the NPCs NAG activity. Although not statistically significant, the exposure to 0.01–1.5 μM MeHg showed a tendency to increase the NAG activity in NPCs.

## 4. Discussion

MeHg is an environmentally found neurotoxicant. Most scientific studies investigating its effects on the CNS have used it in the form of its salt, specifically MeHgCl. However, several studies have shown that under physiological conditions, MeHg is typically bound to a thiol or selenol group [2,3,4,5,6,7]. In this way, here we demonstrated for the first time the effect of physiologically relevant species of MeHg (MeHg–cysteine and MeHg–glutathione) in human MSCs and their derivatives, NPCs. The cell viability findings demonstrated that the cell survival was above 80% after the exposure to MeHg chemical species, indicating that MSCs maintained strong viability even at the highest concentrations of the tested compounds. This result suggests that MSCs have a relative resistance to the toxic effects of MeHg and its cysteine- and glutathione-conjugated variants, at least under the conditions and concentrations evaluated. On the other hand, the exposure to free MeHg significantly decreased the NPCs’ viability. These results suggest that NPCs are more susceptible to cell damage induced by free MeHg, whereas its conjugated forms with cysteine and glutathione appear to have a less pronounced impact. In accordance, Zimmermann et al. [21] observed that free MeHg was more toxic to glioma cells (C6) than the MeHg–cysteine complex.

The antioxidant enzyme activities of MSCs and NPCs after exposure to MeHg and its physiologically relevant species (MeHg–cysteine and MeHg–glutathione) were assessed. The activities of antioxidant enzymes such as SOD, GPx, and GST, as well as GSH concentration, were evaluated as markers of the cellular stress response. Alterations in these enzyme activities and GSH levels may indicate that the cells are undergoing oxidative stress, which can lead to macromolecular damage and ultimately culminate in cell death [18,37]. SOD activity was not altered in the cells after 24 h of exposure to MeHg or its chemical species. This demonstrated that MSCs maintained robust antioxidant activity across all conditions, suggesting their strong inherent defense mechanisms against oxidative damage. In this context, Valle-Prieto and Conget [38] demonstrated that bone marrow human MSCs have a high resistance to oxidative stress-induced death, which correlates with high levels of GSH. Conversely, NPCs displayed a decrease in SOD activity (although not statistically significant), particularly in response to MeHg–glutathione, underscoring their vulnerability to oxidative stress.

The activity of GST and GPx, two critical enzymes involved in detoxification and antioxidant defenses, followed a similar trend. In MSCs, these enzymes remained relatively stable, demonstrating that the cells kept their detoxification capacity even when challenged with a toxicant. However, NPCs exhibited a notable reduction in GST and GPx activities, particularly at higher concentrations of MeHg–glutathione. The impairment of these enzymes in NPCs reinforces the view that these cells could be less equipped to neutralize oxidative and electrophilic stress, making them more susceptible to MeHg-induced damage. Measurements of GSH concentrations revealed that MSCs largely preserved their GSH levels after MeHg exposure, indicating their resilience to this toxicant. In contrast, NPCs experienced a depletion in GSH reserves (although not statistically significant), particularly in response to MeHg–glutathione. In addition, both MSCs and NPCs showed significant reductions in NAG activity when exposed to MeHg–glutathione, but the reduction was more pronounced in NPCs. This decrease in lysosomal function indicates compromised cellular clearance processes [39], particularly in NPCs, which may contribute to MeHg neurotoxicity.

The results obtained from this study highlight significant differences in the response of MSCs and NPCs to exposure to different MeHg chemical species. This variation in response can be attributed to each cell type’s unique cellular characteristics and defense mechanisms. MSCs, for example, demonstrated a higher resistance to the cytotoxic effects of MeHg, regardless of the form in which the MeHg was present (MeHg, MeHg–cysteine, or MeHg–glutathione). This resistance may be due to their inherent immunomodulatory properties and ability to secrete bioactive factors that modulate the inflammatory response and enhance cell survival [40,41,42]. MSCs’ resilience to toxic stressors underlines their potential as therapeutic agents in regenerative medicine [43].

In contrast, NPCs exhibited greater susceptibility to MeHg than MSCs, particularly in its free form. This heightened sensitivity is likely tied to their critical role in the central nervous system, where disruptions can profoundly affect neurogenesis and overall neural function [44,45]. The significant reduction in cell viability observed at higher concentrations of MeHg in NPCs highlights their susceptibility to cellular dysfunction or apoptosis induced by environmental toxins. Given the neurotoxic effects of MeHg, these findings are consistent with the literature, which often associates MeHg exposure with neuronal damage and central nervous system impairments [37,46,47].

Overall, these results underscore the importance of studying different cell lines when investigating the mechanisms of MeHg toxicity. The results highlight the differences in biochemical responses between MSCs and NPCs, revealing the greater vulnerability of NPCs to oxidative stress induced by MeHg, especially in its form conjugated with glutathione. These findings provide important insights into the cellular defense mechanisms involved in organic mercury toxicity. On the other hand, the observed impact on NPCs may help explain the heightened vulnerability of children—and their immature neurons, such as neural progenitor cells that originated in the brain—to heavy metal exposure, reinforcing the need for strict environmental regulation.

## 5. Conclusions

The biochemical analyses, combined with cell viability data, provided a comprehensive understanding of the distinct susceptibilities of MSCs and NPCs to MeHg species. The greater resilience of MSCs can be attributed to their robust antioxidant systems and detoxification pathways, making them more suitable for therapeutic applications in environments exposed to oxidative stress. On the other hand, the pronounced vulnerability of NPCs, particularly to MeHg–glutathione, reinforces the neurotoxic potential of methylmercury, highlighting the importance of protecting immature neural cells from environmental toxins. The absence of MeHg–cysteine effects was an unexpected result, since the studies show that, usually, MeHg gets inside the cells as a mimic of the amino acid methionine, bound to a Cys molecule. More studies are necessary to understand this result. In addition, certain limitations should be acknowledged. Because we are working with a primary cell culture, which is inherently more variable, the limited sample size may have contributed to the absence of observable effects in some assays.

## Data Availability

The data generated in this study are presented in the manuscript and the Supplementary Materials.

## Supplementary Materials

The following supporting information can be downloaded at: https://www.mdpi.com/article/10.3390/metabo15120794/s1, Figure S1: Adipogenic differentiation; Figure S2: Osteogenic differentiation; Figure S3: Chondrogenic differentiation; Figure S4: Formation of the neurosphere; Figure S5: Microscopic view of the MSCs and NPCs; Figure S6: Histogram representing the isotypic control (green) and the degree of expression of the cell markers.
