# Supplementary material for: Effects of Physiologically Relevant Species of Organic Mercury on Mesenchymal Stem Cells and Neural Precursor Cells
Source: Metabolites. 2025 Dec 14;15(12):794. doi: 10.3390/metabo15120794 (PMC12735294; doi:10.3390/metabo15120794)
Supplement: Supplementary file 1 [file metabolites-15-00794-s001.zip › metabolites-3923696-supplementary.pdf]

Supplementary Materials

Effects of Physiologically Relevant Species of Organic Mercury on Mesenchymal Stem Cells and Neural Precursor Cells

Juliane Hostert [1,2], Nathalia Kirsten [1,2], Larissa Lührs [1,2], Ana Carolina Irioda [1], Izonete Cristina Guiloski [1,2], Katherine Athayde Teixeira de Carvalho [1,2] and Cláudia Sirlene Oliveira [1,2,*]

[1] Instituto de Pesquisa Pelé Pequeno Príncipe, Curitiba 80250-060, PR, Brazil

[2] Faculdades Pequeno Príncipe, Curitiba 80230-020, PR, Brazil

* Correspondence: claudia.sirlene@professor.fpp.edu.br

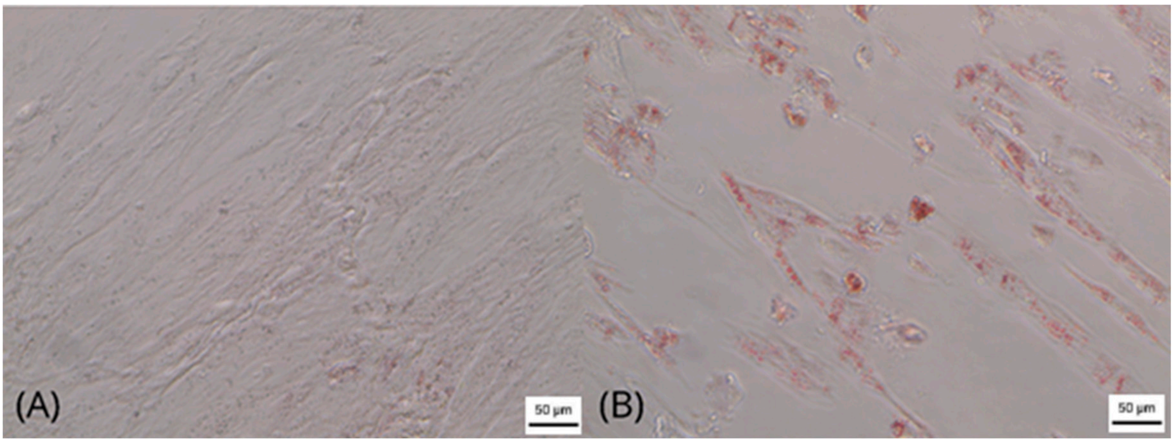

Figure S1 of supplementary material: Adipogenic differentiation. (A) Undifferentiated MSCs. (B) MSCs subjected to adipogenic differentiation, showing staining of lipid vacuoles with Oil Red O. Image obtained using an inversion optical microscope at 100x magnification (Axio Vert A1, Car Zeiss, Oberkochen, Germany)..

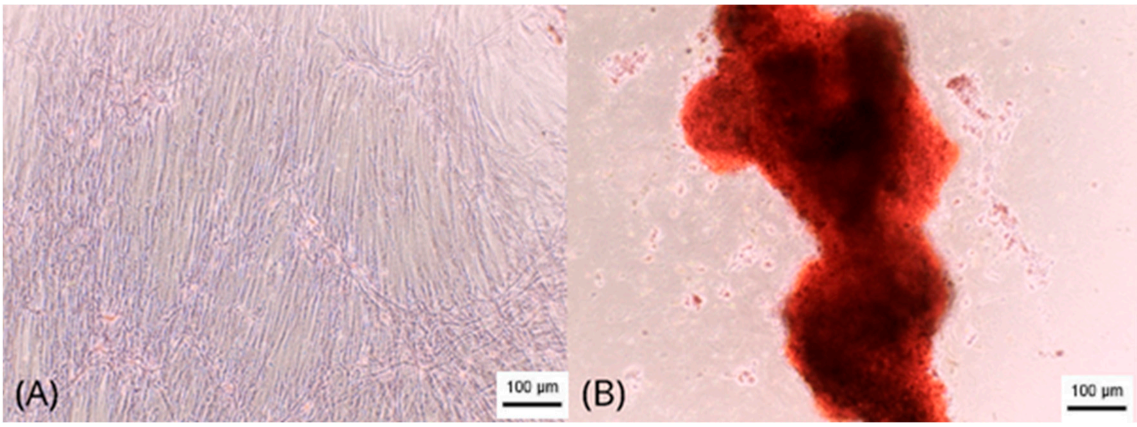

Figure S2 of supplementary material: Osteogenic differentiation. (A) Undifferentiated MSCs. (B) MSCs subjected to osteogenic differentiation showing mineralization stained by alizarin red. Image obtained using an inversion optical microscope at 100x magnification (Axio Vert A1, Car Zeiss, Oberkochen, Germany).

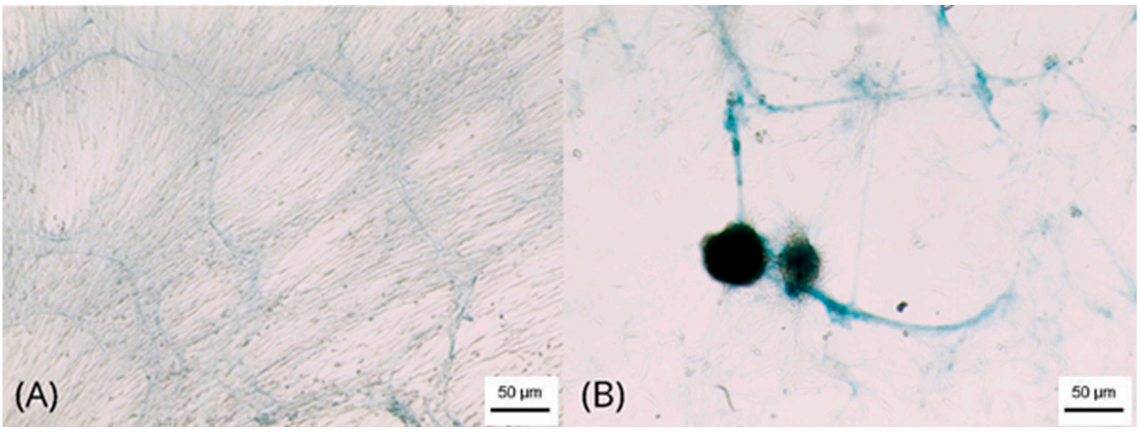

Figure S3 of supplementary material: Chondrogenic differentiation. (A) Undifferentiated MSCs. (B) MSCs subjected to chondrocytes differentiation showing production of proteoglycans, which are stained with Alcian blue. Image obtained using an inversion optical microscope at 100x magnification (Axio Vert A1, Car Zeiss, Oberkochen, Germany).

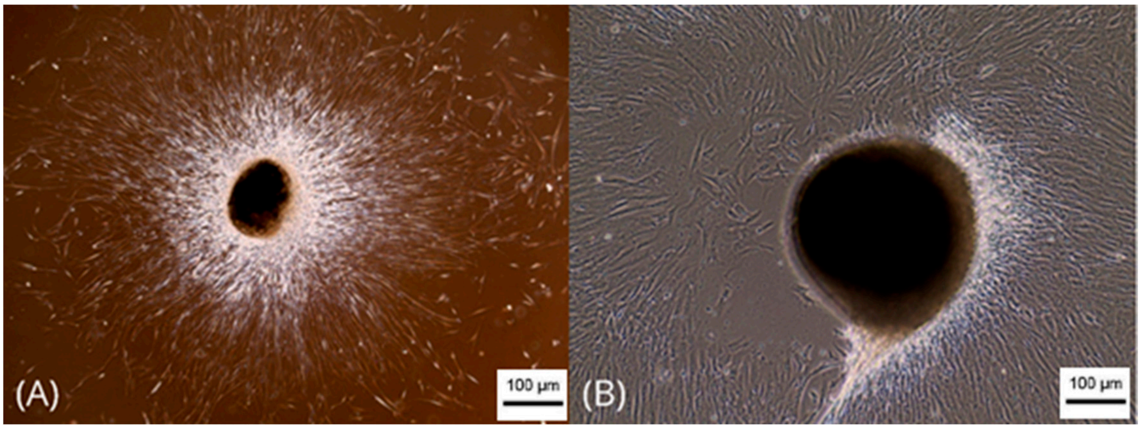

Figure S4 of supplementary material: (A) Formation of the neurosphere. (B) Neurosphere derived from MSCs. Image obtained using an inversion optical microscope at 100x magnification (Axio Vert A1, Car Zeiss, Oberkochen, Germany).

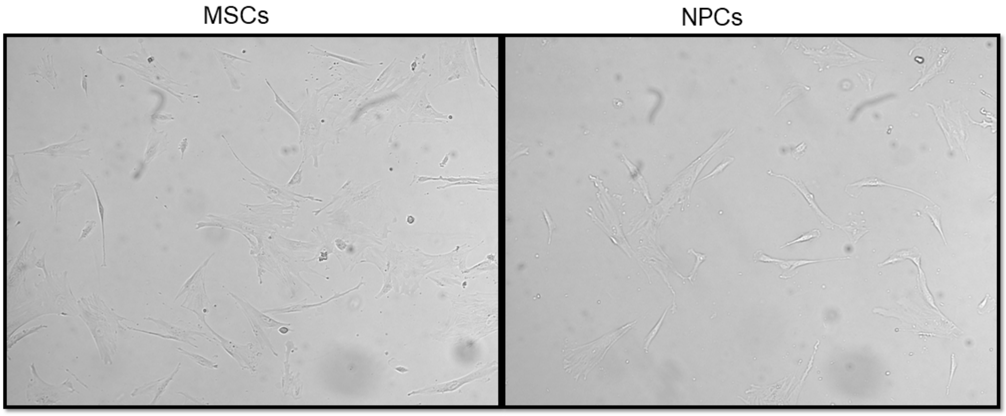

Figure S5 of supplementary material: Microscopic view of the MSCs and NPCs. Image obtained using an inversion optical microscope at 10x magnification (Axio Vert A1, Car Zeiss, Oberkochen, Germany).

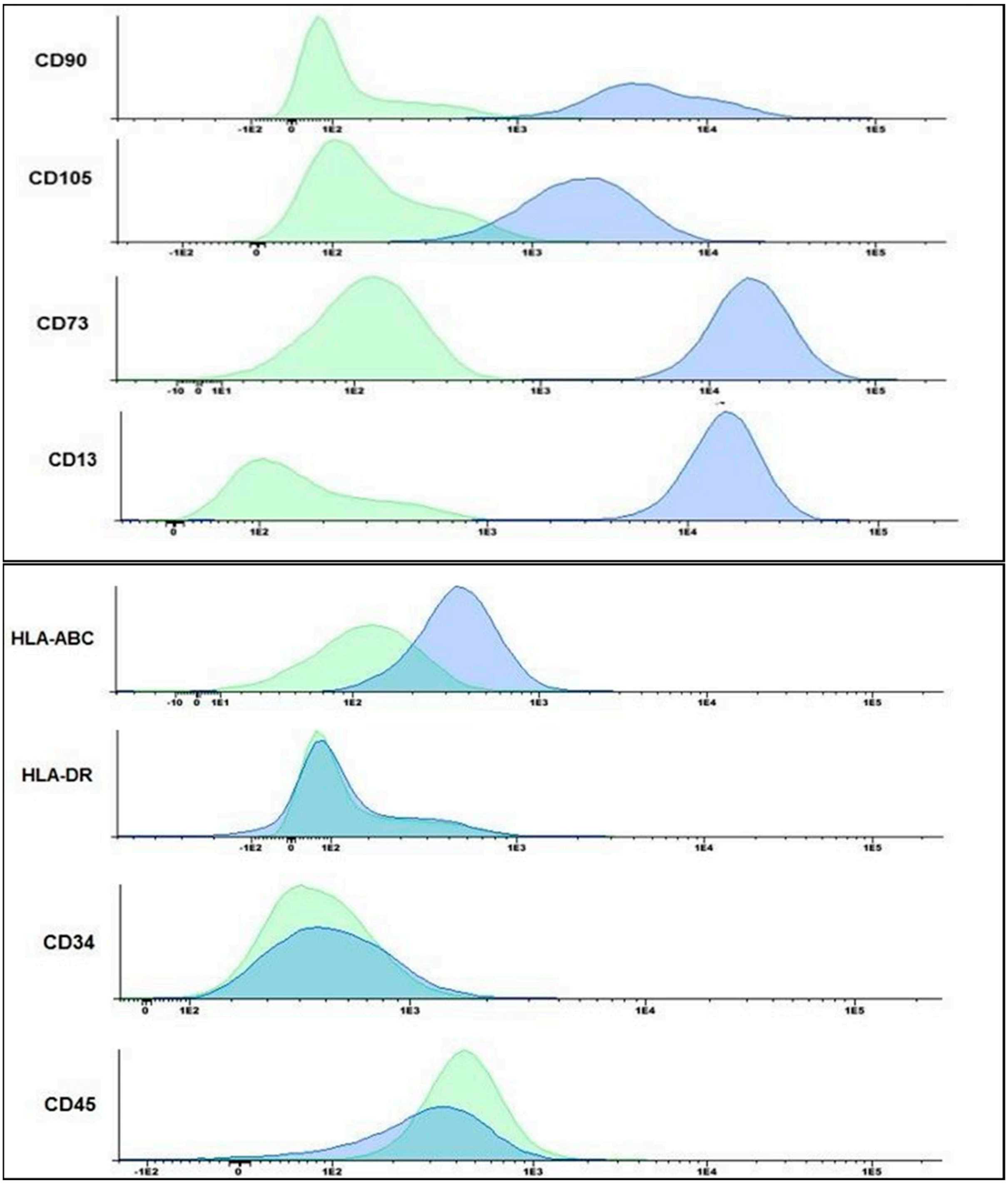

Figure S6 of supplementary material: Histogram representing the isotypic control (green) and the degree of expression of the markers. The positive expression is located to the right of the isotypic control histogram.
